# Genome-Wide Analysis of the Oat (*Avena sativa*) *HSP90* Gene Family Reveals Its Identification, Evolution, and Response to Abiotic Stress

**DOI:** 10.3390/ijms25042305

**Published:** 2024-02-15

**Authors:** Jinghan Peng, Siyu Liu, Jiqiang Wu, Tianqi Liu, Boyang Liu, Yi Xiong, Junming Zhao, Minghong You, Xiong Lei, Xiao Ma

**Affiliations:** 1 College of Grassland Science and Technology, Sichuan Agricultural University, Chengdu 611130, China; 2 Sichuan Academy of Grassland Science, Chengdu 610097, China

**Keywords:** oat, heat shock protein, HSP90, phylogenetic analysis, expression pattern

## Abstract

Oats (*Avena sativa*) are an important cereal crop and cool-season forage worldwide. Heat shock protein 90 (HSP90) is a protein ubiquitously expressed in response to heat stress in almost all plants. To date, the *HSP90* gene family has not been comprehensively reported in oats. Herein, we have identified twenty *HSP90* genes in oats and elucidated their evolutionary pathways and responses to five abiotic stresses. The gene structure and motif analyses demonstrated consistency across the phylogenetic tree branches, and the groups exhibited relative structural conservation. Additionally, we identified ten pairs of segmentally duplicated genes in oats. Interspecies synteny analysis and orthologous gene identification indicated that oats share a significant number of orthologous genes with their ancestral species; this implies that the expansion of the oat *HSP90* gene family may have occurred through oat polyploidization and large fragment duplication. The analysis of cis-acting elements revealed their influential role in the expression pattern of HSP90 genes under abiotic stresses. Analysis of oat gene expression under high-temperature, salt, cadmium (Cd), polyethylene glycol (PEG), and abscisic acid (ABA) stresses demonstrated that most AsHSP90 genes were significantly up-regulated by heat stress, particularly *AsHSP90-7*, *AsHSP90-8*, and *AsHSP90-9*. This study offers new insights into the amplification and evolutionary processes of the AsHSP90 protein, as well as its potential role in response to abiotic stresses. Furthermore, it lays the groundwork for understanding oat adaptation to abiotic stress, contributing to research and applications in plant breeding.

## 1. Introduction

The quality of life of terrestrial organisms on Earth is increasingly impacted by climate change [1]. As sessile organisms, plants are particularly susceptible to environmental stresses such as drought, salinity, cold, and heat during growth and development [2]. Recently, heat stress has become a significant abiotic stresses affecting normal plant growth and development due to global warming, increased droughts, and extreme weather conditions [3]. In particular, prolonged growth at high temperatures severely inhibits starch synthesis and carbon assimilation, leading to a reduction in average yields and posing a major challenge to food security [4,5]. Furthermore, the exposure of plants to premature high temperatures during unsuitable phenological periods makes them more susceptible to pathogen infection, potentially affecting the quality of crop production [6].

Plants have developed regulatory mechanisms to cope with heat stress and thousands of genes are involved during the evolution of their long-term adaptation [7]. Among them, the heat shock protein (HSP) is one of the best-characterized genes and plays a significant role in regulating responses to heat stress. Generally, HSPs can be categorized into HSP20, HSP60, HSP70/DnaK, HSP90, and HSP100/ClpB families according to their molecular weight and sequence homogeneity [8], of which HSP90s are highly conserved in molecular evolution and an abundant family of ATP-dependent molecular chaperone proteins in prokaryotes and higher eukaryotes [9], which are broadly distributed in the cytoplasm, chloroplasts, mitochondria, and endoplasmic reticulum, accounting for 1–2% of total cellular proteins [10,11]. *HSP90* generally contains three structural domains: the N-terminal ATP-binding domain, the M domain, and the C-terminal substrate-binding domain [12]. *HSP90* features an unconventional Bergerat ATP-binding fold and is part of the GHKL superfamily [13]. According to previous studies, HSP90 proteins often have dual functions: HSP90s are involved in regulating and maintaining the conformation of various proteins and assisting normal cell survival under stress on the one hand [14], and act as negative feedback regulators of heat stress responses on the other [15]. *HSP90*, along with other molecular chaperones, provides a mechanism to promote protein folding [12], prevents protein aggregation, and facilitates the refolding of inactivated proteins, thereby increasing the resistance of certain cells [14]. *HSP90* expression is up-regulated when plants are stressed; it then associates with nonprotein substances to enable the repair of deformed proteins [16]. Besides its importance for protein folding, a function of *HSP90*s as negative regulators for heat stress transcription factors (Hsfs) has been proposed for Hsf1, whose activities in facilitating downstream gene expression are tightly regulated [7].

Recently, *HSP90*s have been identified and found in many plants, including seven members of the *HSP90* members in *Arabidopsis thaliana* [17], eight in *Brachypodium distachyon* [18], twenty-one in *Nicotiana tabacum* [19], and eight in *Perennial Ryegrass* [20], and directly or indirectly implicated in a host of physiological processes ranging from plant growth and development to abiotic and biotic stress responses [8]. Overexpression of *AtHSP90-2*, *AtHSP90-5*, and *AtHSP90-7* in *Arabidopsis thaliana* reduces tolerance to salt and drought stress but increases tolerance to high Ca^2+^ concentrations [21]. Both tobacco and *Arabidopsis* species have HSP90 members that convey resistance to pathogens by counteracting the response of signaling receptor R proteins from pathogens [22,23]. Additionally, up-regulation of the *VvHsp901a* gene was delayed when grape plants were subjected to drought and high-temperature stresses [24]. The HSP90 protein also plays a role in plant growth and developmental processes, and its mediated distribution of PIN1 regulates the distribution of auxin signaling, thereby promoting plant growth and development [25].

Oats (*Avena sativa* L.) are a global crop and one of the richest sources of protein, fat, and β-glucan among all cereals, with a low carbon footprint [26,27]. Also, oats are a widely grown annual forage worldwide and an important source of high-quality pasture for livestock [26]. Oats are a cool-season crop that is suitable for growing in humid environments [28], but due to severe global warming, temperature conditions are quickly met, which may lead to shorter growing periods, smaller plants, and lower yields in oats. Therefore, it is of great significance to understand and reveal the molecular members (e.g., HSP90s) that contribute to high temperature tolerance in oats. To date, a systematic and comprehensive study of the *HSP90* gene family in oats has not yet been reported, and the assembly of a high-quality oat genome provides the necessary information to characterize *HSP90*s at the genome-wide level [29]. In this study, we characterized the *HSP90* gene within the oat genome, including gene sequence and homology analyses, and described the evolutionary pathway of the HSP90 gene in oats. Additionally, we analyzed oat HSP90 gene expression under high-temperature, salt, cadmium (Cd), polyethylene glycol (PEG), and abscisic acid (ABA) stresses using Quantitative Real-Time PCR. Our study provides a new avenue for molecular breeding in oats, contributes to a better understanding of the heat tolerance response of oats under high-temperature conditions, and lays the foundation for future studies on the function of the AsHSP90 protein in oat stress tolerance.

## 2. Results

### 2.1. Identification of Oat HSP90 Genes and Chromosomal Distribution

A total of 20 *HSP90* gene family members has been identified in *A. sativa* (Table 1). AsHSP90 proteins were renamed *AsHSP90-1* to *AsHSP90-20* based on their molecular weight. The obtained *AsHSP90* sequences varied in length ranging from 627 to 809 amino acids, with pI values ranging from 4.6 to 5.09 and molecular weights ranging from 71,881.4 kd to 92,623.6 kd. AsHSP90 proteins are predominantly cytoplasmic and chloroplastic, except for *AsHSP90-5*, *AsHSP90-10*, *AsHSP90-1*, *AsHSP90-15*, and *AsHSP90-20*, which are localized in the nucleus and ER, respectively.

**Figure 1:**
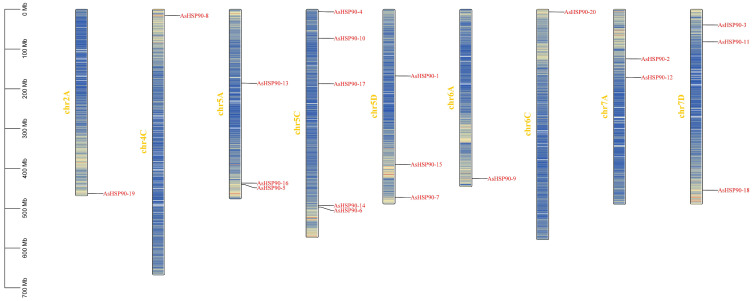
Chromosomal localization of members of the oat *HSP90* gene family. Blue to yellow colors within the chromosomes indicate increased gene density. Chromosome numbers are shown at the right of the vertical bar; gene locations are shown at the left of the vertical bar.

Members of the *AsHSP90* gene family are distributed across nine chromosomes and most of them are located in positions of high gene density (Figure 1), and most *AsHSP90* genes are located on chr5 (chr5A, chr5C, chr5D). chr5 contains the most *AsHSP90* genes, although it is not the longest chromosome. Gene clusters can be observed on chr5A, chr5C. This uneven distribution might be the result of uneven replication of oat chromosome segments. In addition, it was noted that relatively less *AsHSP90* genes were located on chr2A, chr6A, and chr6C.

### 2.2. Phylogenetic Analysis of AsHSP90 Genes

**Figure 2:**
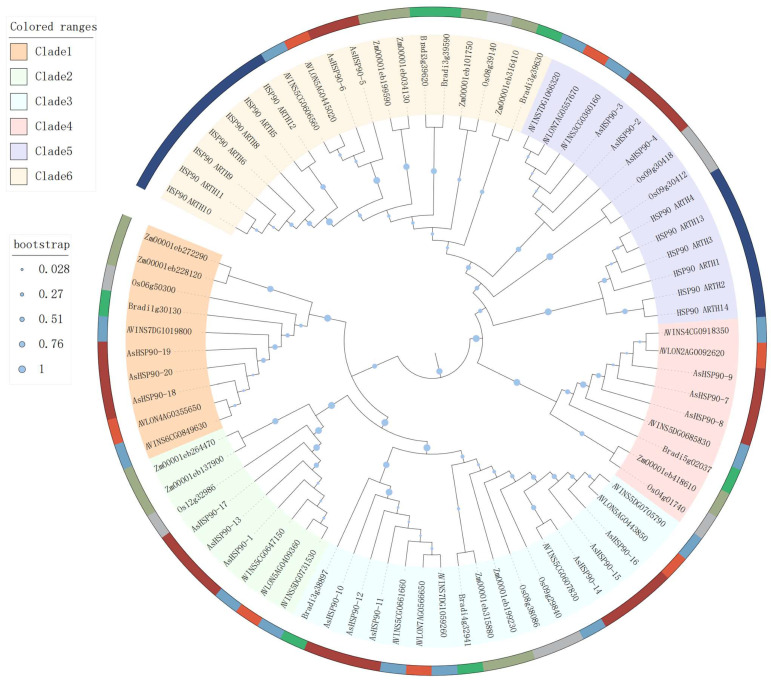
Phylogenetic tree analysis of the HSP90 proteins from *A. sativa*, *A. insularis*, *A. longiglumis*, *Arabidopsis thaliana*, *Brachypodium disachyon*, rice, and maize. The HSP90s were divided into six clades (Clades 1–6) based on the clustering of the protein sequence. The proteins from *A. sativa*, *A. insularis*, *A. longiglumis*, *Arabidopsis thaliana*, *Brachypodium disachyon*, rice, and maize are presented in brown, blue, dark orange, dark blue, green, gray, and dark green, respectively.

In order to understand the evolutionary relationships of the *HSP90* members, a phylogenetic tree including seven *Arabidopsis* HSP90s, eight rice HSP90s, eight *Zea mays* HSP90s, nine *Brachypodium disachyon* HSP90s, seven *A. insularis* HSP90s, and thirteen *A. longiglumis* HSP90s was constructed using the maximum likelihood (ML) method with MEGA7.0 software (Figure 2). All HSP90 protein sequences were categorized into six clades (Clades 1, 2, 3, 4, 5, and 6). Clade 6 (19 members) had the most number of members, followed by Clade 3 (18 members). Seven and six species were identified in Clade 6 and Clade 3, respectively. In the phylogenetic tree, all oat HSP90 genes showed a closer evolutionary relationship with members of *A. insularis* and *A. longiglumis*. Interestingly, all *Arabidopsis* HSP90s genes were assigned to Clade 6, which may be related to the fact that it is the only dicotyledonous plant in the phylogenetic tree.

### 2.3. Motif Pattern and Gene Structure Analyses of AsHSP90 Members

**Figure 3:**
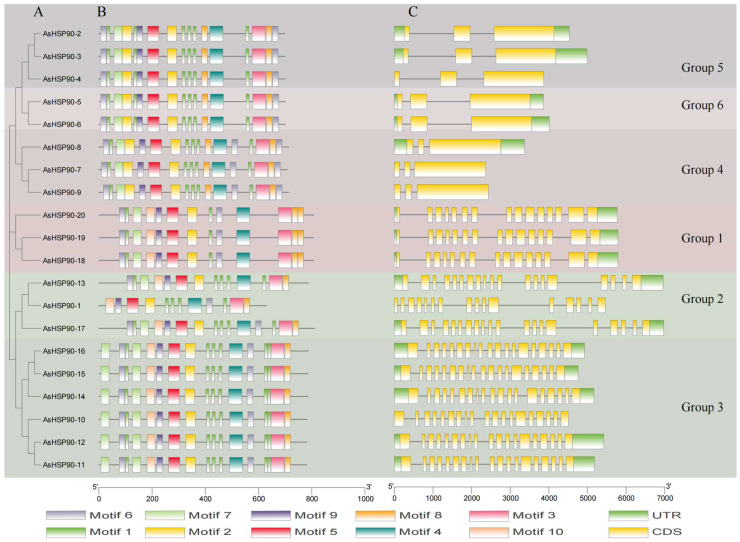
Phylogenetic tree, motif analysis, and gene structure of *AsHSP90*: (**A**) Phylogenetic tree analysis of the AsHSP90 protein. (**B**) Motif composition of *AsHSP90*. (**C**) Gene structure of the *AsHSP90* genes in oats.

The evolution of the oat HSP90 gene family was revealed by analyzing the gene structure and motifs of the *AsHSP90* genes. The 20 AsHSP90s proteins could be placed in six groups according to a constructed simplified phylogenetic tree. Of these, Groups 3 and 6 had the most and least members, with six and two, respectively (Figure 3A). All of these genes had between 2 and 18 introns. However, Groups 4, 5, and 6 contained 2 introns, while the remaining three groups contained 15–18 introns (Figure 3C). In addition, genes on the same branch of the evolutionary tree are similar in structure, and their CDSs have similar numbers of introns. There was little variation in the location and length of the introns within the groups, but significant variation between the groups.

To better understand the structural quality of the AsHSP90 protein, we identified 10 conserved motifs in the protein using the MEME [30] motif search tool and explored the distribution of these conserved motifs in the AsHSP90 protein (Figure 3B, Supplementary Figure S1). The results of this study showed that the 10 most conserved motifs detected contained 12–50 amino acids. Among them, motif 1 had the lowest amino acid content, with 12 amino acids. In addition, most of the genes consisted of 10 conserved motifs. Similar genes had similar motifs, which suggests that the *AsHSP90* gene family has similar functions. Overall, the *HSP90* gene family in oats is highly conserved, with few conserved motifs lost during evolution.

### 2.4. Duplication Analysis of AsHSP90 Members

**Figure 4:**
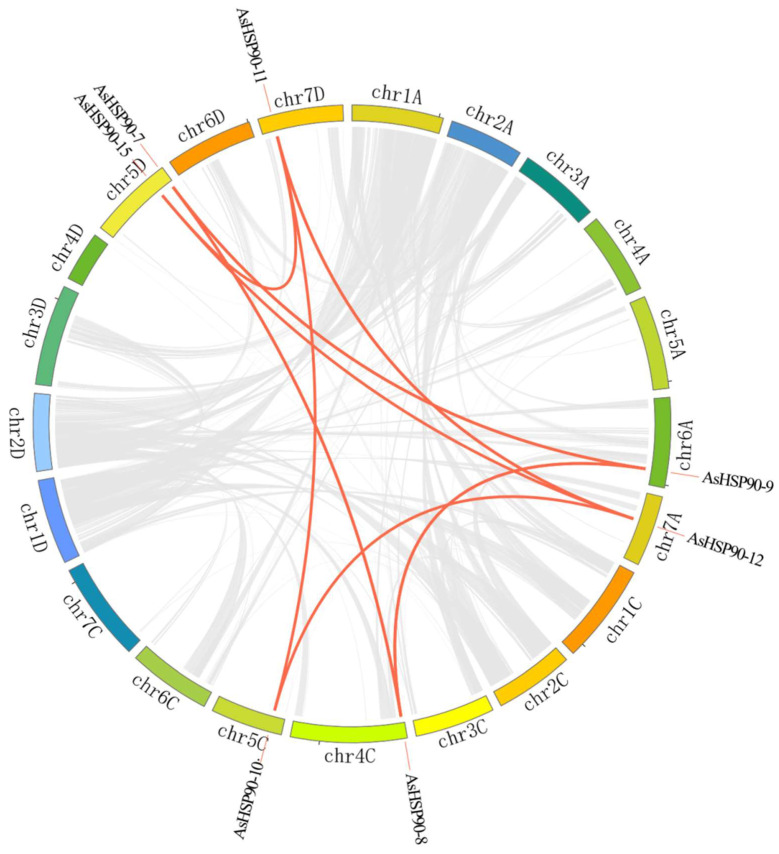
Gene duplications of the oat HSP90 gene family. Red lines indicate duplicated gene pairs in *AsHSP90*, and gray lines indicate co-linear gene pairs in the genome.

Gene duplication events in the *AsHSP90* gene were analyzed using MCScanX [31]. In total, there were 10 pairs of duplication genes among *HSP90* genes (Figure 4 and Table 2). All duplicates were from Group 3 and Group 4, and the majority of them were located at the end of the chromosome. These 10 pairs of genes were defined as WGD/segmental duplicates and inter-chromosomal. Some *AsHSP90* genes had undergone more than one duplication. In addition, we calculated the non-synonymous (Ka) and synonymous (Ks) substitution rates, as well as Ka/Ks ratios (Table 2), to capture the evolutionary dynamics of the ASHSP90 protein coding sequence. Of these, 10 pairs of duplication genes had Ka/Ks values of <1, suggesting that these genes had undergone primarily purifying selection.

### 2.5. Collinearity Analysis of HSP90 Genes

**Figure 5:**
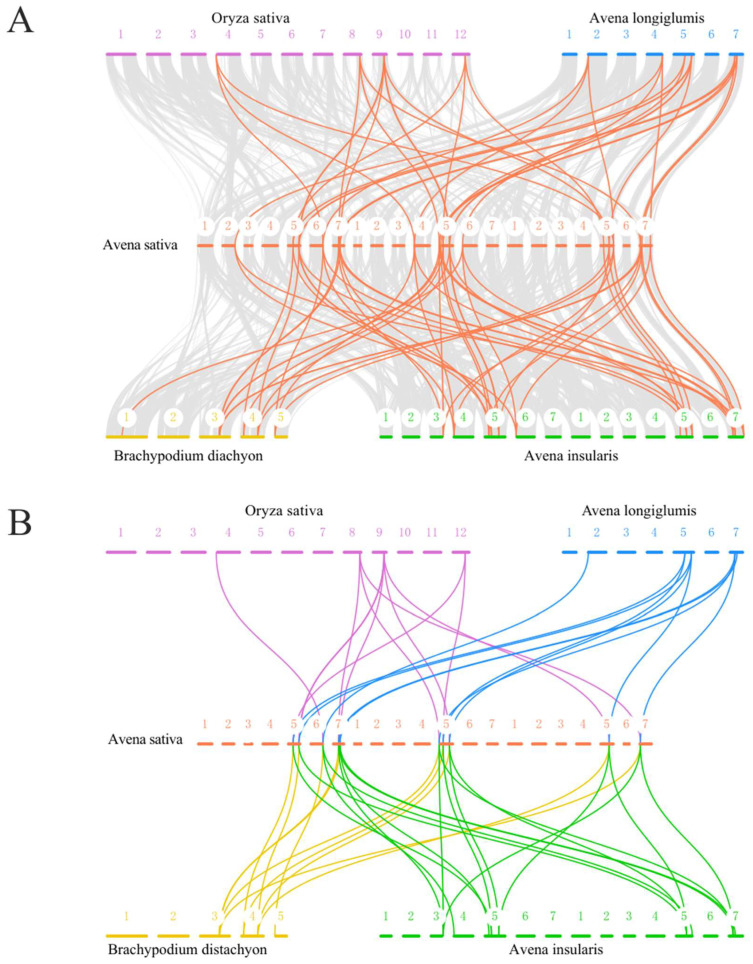
Synteny analysis of *AsHSP90*s in *A. sativa* and four representative plants. (**A**) All AsHSP90 synteny genes in oats and in *Oryza sativa*, *A. longiglumis*, *Brachypodium distachyon*, and *A. insularis* are indicated by red lines. The synteny blocks in the oats and the other species are shown in gray lines. (**B**) The nine *AsHSP90* genes with covariance in the four species are shown as purple, blue, yellow, and green lines.

To explore the evolutionary relationship between the oat *AsHSP90* gene and other species, synteny analyses were performed on four representative plants, including two possible ancestral species of oats (*A. insularis* and *A. longiglumis*) and two monocotyledons (*Oryza sativa* and *Brachypodium distachyon*). Thirty-seven and nineteen *AsHSP90* syntenic gene pairs were identified in *A. insularis* and *A. longiglumis*, respectively, and fifteen syntenic gene pairs each were identified in *Oryza sativa* and *Brachypodium distachyon* (Figure 5A, Supplementary Table S1). Nine *AsHSP90*s (*AsHSP90-2*, *AsHSP90-3*, *AsHSP90-4, AsHSP90-12*, *AsHSP90-13*, *AsHSP90-14*, *AsHSP90-15*, *AsHSP90-16*, and *AsHSP90-17*) are present as syntenic genes in these four species (Figure 5B). In addition, syntenic genes of the oat HSP90 gene Group 1 (*AsHSP90-1*8, *AsHSP90-19*, and *AsHSP-20*) were not found in either rice or *Brachypodium distachyon*, but their respective syntenic genes were found in *A. Insularis* and *A. longiglumis*. On this basis, we separately detected *AsHSP90* orthologs in rice, *A. longiglumis*, *Brachypodium distachyon*, and *A. insularis* with OrthoFinder (Supplementary Table S2). *A. longiglumis* and *A. insularis* contained 7 and 12 orthologous genes, respectively, and *Brachypodium distachyon* contained 3. Interestingly, two and one genes in *A. insularis* and *A. longiglumis*, respectively, showed to be orthologous to oat *AsHSP90* gene Group 1 (*AsHSP90-18*, *AsHSP90-19*, and *AsHSP-20*), and all of these genes were from different sub-genomes. In addition, all three genes belong to the same group and none of them were involved in duplication events, which may suggest that these three genes are more conserved during the evolutionary process.

### 2.6. Cis-Element Analysis of AsHSP90 Gene Promoters

**Figure 6:**
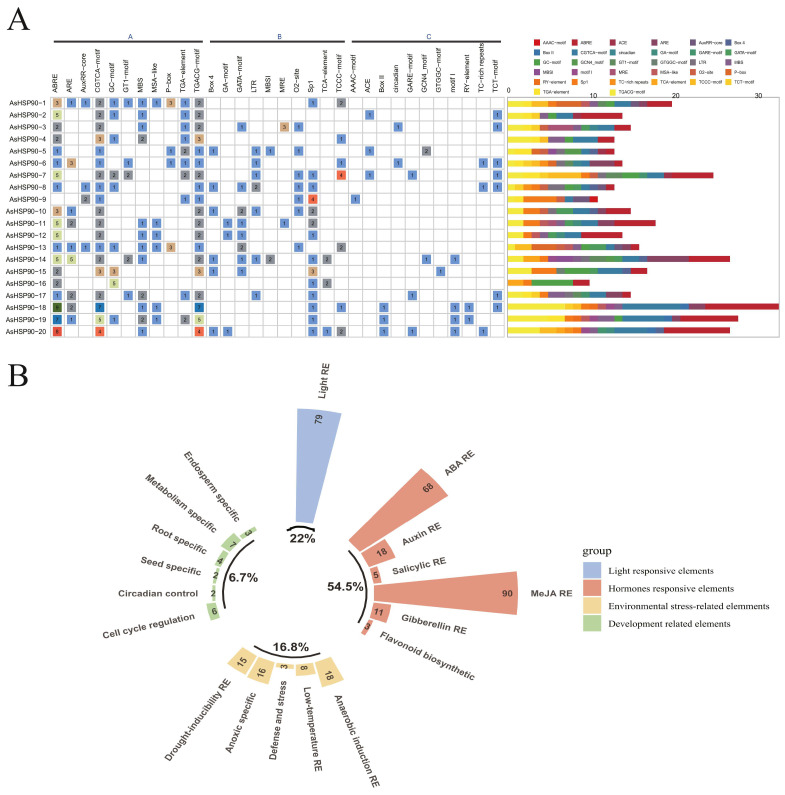
Distribution of cis-elements upstream of oat *AsHSP90*: (**A**) Distribution of types and numbers of cis-elements in the promoter of the oat *AsHSP90* genes. (**B**) Classification and proportion of cis-elements in the *AsHSP90* gene.

Sequences 1500 bp upstream of the promoter of each *AsHSP90* gene were isolated from the oat genome, and cis-acting elements were predicted using PlantCARE [32] to characterize the expression of each *AsHSP90* gene. A total of 32 cis-acting elements were analyzed (Figure 6, Supplementary Table S3). *AsHSP90-18* has the highest number of homeopathic acting elements and *AsHSP90-16* has the lowest number of homeopathic acting elements. The 20 *AsHSP90* promoter regions involved hormone-responsive elements, light-responsive elements, environment-responsive elements, and developmentally relevant elements (Figure 6B). These elements mainly responded to hormonal and abiotic stresses. Interestingly, there are two cis-acting regulatory elements involved in circadian control in *AsHSP90-3* and *AsHSP90-6*. Furthermore, a total of five cis-elements related to salicylic acid responsiveness were found in *AsHSP90-14*, *AsHSP90-16*, and *AsHSP90-20*.

### 2.7. Expression Analysis of AsHSP90s in Oats under Five Abiotic Stresses

**Figure 7:**
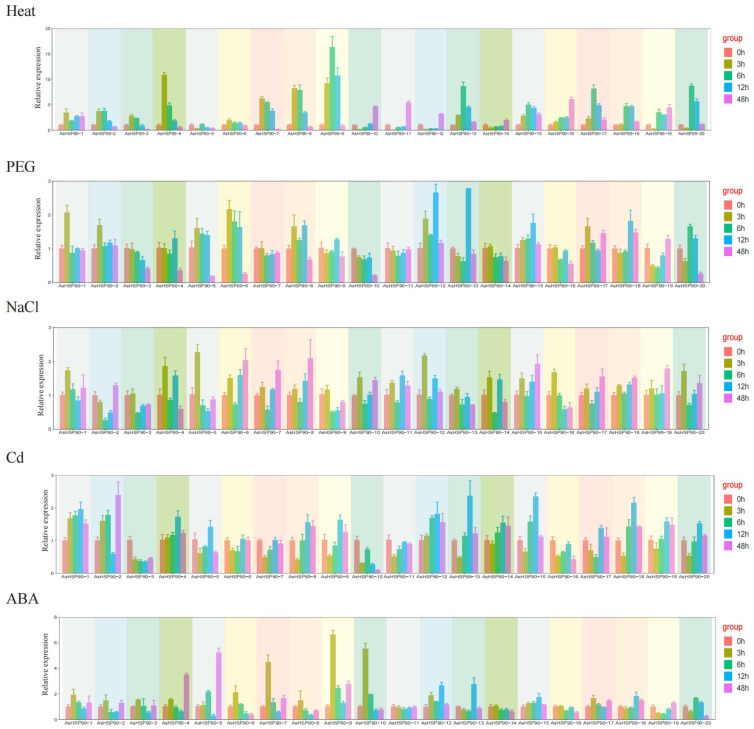
The relative expression of 20 *AsHSP90* genes in oat leaves was detected with qRT-PCR after treatments of 0 h (CK), 3 h, 6 h, 12 h, and 48 h under different abiotic stresses. Error bars indicate the standard error (SE) between three replicates.

To analyze the expression pattern of *AsHSP90* under several different abiotic stresses, 20 AsHSP90 proteins were analyzed using qRT-PCR. As shown in Figure 7, different expression patterns of 20 *AsHSP90*s were observed under heat, drought, salt, Cd, and ABA stresses. Almost all AsHSP90 members were involved in expression under different abiotic stresses. Among the five treatments, heat stress elicited the most pronounced stress response, with the average expression of AsHSP90 genes being two–eight times higher than that under the other four abiotic stresses. AsHSP90-7, AsHSP90-8, and AsHSP90-9 exhibited prominent expression in response to heat stress, while other members also demonstrated varying levels of transcriptional activation.

Under the drought treatment (PEG treatment simulation), AsHSP90-12 and *AsHSP90-13* were significantly expressed at 12 h, but their expression decreased with increasing exposure time. *AsHSP90-1*, *AsHSP90-5*, and *AsHSP90-6* were also observed to be expressed at the 6 h point. Furthermore, the expression of *AsHSP90-3*, *AsHSP90-4*, *AsHSP90-5*, *AsHSP90-6*, *AsHSP90-10*, and *AsHSP90-20* was significantly reduced at 48 h. Under NaCl treatment, the expression of all members except *AsHSP90-3* increased at 6 h and then decreased and then increased with longer exposure time.

Under Cd treatment, *AsHSP90-13*, *AsHSP90-15*, and *AsHSP90-18* were significantly expressed at 12 h, and *AsHSP90-2* was significantly expressed at 48 h. The expression of other *AsHSP90*s showed a fluctuating pattern, suggesting that their expression may be influenced by the duration of Cd treatment. *AsHSP90-7*, *AsHSP90-9*, and *AsHSP90-10* were significantly expressed under ABA treatment at 6 h; *AsHSP90-4* and *AsHSP90-5* were significantly up-regulated at 48 h and continued to be up-regulated, while the expression of the other members was not significant.

In general, Group 5 (*AsHSP90-7*, *AsHSP90-8*, and *AsHSP90-9*) in the oat *AsHSP90* gene was induced under all five stresses. Among all of the abiotic stresses, the highest expression of *AsHSP90-9* was found after 12 h of heat stress.

### 2.8. Three-Dimensional Structure Prediction and Protein–Protein Interaction Network

Three-dimensional protein structures of the AsHSP90s were performed by the SWISS-MODEL [33] server and model generation was performed via PyMOL (Supplementary Figure S2). All targets had greater than 30% identity with the template, which is a threshold that is a sign of successful modeling (advances in homology protein structure modeling). The QMEAN score values of the models varied between 0.64 and 0.76, which indicated that all of the models were of better quality, while the GMQE values ranged from 0.54 to 0.76. Meanwhile, out of the 20 models, 16 models were hetero-trimer states (Supplementary Table S4).

The protein–protein interaction network was further analyzed to detect interactions between AsHSP90 and related proteins. Seven other proteins were found to interact with AsHSP90 (Supplementary Figure S3), potentially being jointly involved in certain biological processes. Protein nodes were manually rearranged based on their degree of interaction. Proteins positioned in the inner circle of the layout exhibit a higher degree of interaction. In addition to AsHSP90, proteins A0A3B5YXX4 (HSP70), A0A3B6EMP3 (calreticulin), and A0A3B6H015 (calreticulin) also exhibit strong associations with AsHSP90 proteins.

## 3. Discussion

Heat stress protein 90 (HSP90) is a highly conserved molecular chaperone within the HSP family. HSP90 proteins are rapidly synthesized in response to heat stress treatments, serving to counteract the damage caused to plants by high temperatures [10]. Here, we identified twenty HSP90 genes and assigned them to six clades. Furthermore, the number of oat HSP90 genes was higher than in some previously studied species, such as the 7 in *A. thaliana* [17] and 10 in *populus* [34], a phenomenon largely attributable to hexaploidy. These *AsHSP90* genes may play a significant role in the physiological maintenance of oats, allowing them to survive high temperatures and other abiotic stress environments. The *HSP90* family of proteins exhibits varied biophysical properties, indicative of a wide diversity among its members. This diversity lays the groundwork for further studies on the function of *HSP90* genes. In this study, the *AsHSP90* gene sequence displayed an isoelectric point (pI) ranging from 4.60 to 5.09 and was acidic, consistent with previous studies on the *HSP90* gene in *perennial ryegrass* [20]. Additionally, we observed that the *AsHSP90* gene is unevenly distributed in oats, predominantly in regions of high gene density on the chromosomes. This distributional characteristic may be linked to the uneven replication of oat chromosome segments [29].

Oats undergo complex polyploidization events and frequent translocations among subgenomes, which provide a good opportunity to study gene family formation and expansion [29,35]. Gene duplication events are thought to be an important mechanism for increasing gene family diversity [36]. Plant evolution is usually accompanied by gene fragment duplication events, considered as one of the main drivers of the expansion of plant gene families. The oat genome possesses a mosaic chromosome structure, and the chromosomal rearrangements it undergoes often result in the duplication of gene family members [29]. In this study, 10 pairs of segmental duplication genes were identified in the *AsHSP90* gene family. These genes belong to Group 3 and Group 4, comprising homologous genes with similar structural domains. Chromosomal polyploidy also significantly contributes to the expansion of the number of gene families in plants. The “A” subgenome and “CD” subgenome in oats originated from *A. insularis* and *A. longiglumis*, respectively [35]. Also, 13 and 7 HSP90 genes were identified from *A. insularis* and *A. longiglumis*, the probable ancestral species of oats, respectively, and the total number of these genes is consistent with the number of *AsHSP90* genes. Thus, chromosome doubling is likely responsible for AsHSP90 amplification in oats. This finding is consistent with the fact that *A. insularis* and *A. longiglumis* hybridized to form a heterozygous hexaploid. Furthermore, synteny analysis and orthologous gene identification reveal that not all oat *AsHSP90* genes have direct homologs in the ancestral species. For example, the *AsHSP90-9* gene located in the “A” subgenome lacks orthologous genes in *A. insularis*, whereas *AsHSP90-7* and *AsHSP90-8*, which are part of Group 3 in the “CD” subgenome, possess orthologous genes in *A. longiglumis* (Supplementary Table S2). Additionally, there is a gene duplication event between *AsHSP90-7*, *AsHSP90-8*, and *AsHSP90-9*, suggesting that *AsHSP90-9* may be a paralogous gene resulting from this duplication. This implies that the formation of paralogous genes also contributes to the expansion of the *AsHSP90* gene family. In conclusion, the formation of the *AsHSP90* gene family in oats may have arisen mainly through gene duplication after polyploidy and divergence.

Phylogenetic analyses can help to understand evolutionary relationships between species and ermine homology between and within species [37]. In this study, a phylogenetic tree was constructed using the protein sequences of the HSP90 gene from seven monocotyledonous and one dicotyledonous plant species. Based on the phylogenetic analysis, it can be categorized into six distinct clades, wherein all *Arabidopsis* HSP90 genes cluster in Clade 5, which may be attributed to *Arabidopsis* being the sole dicotyledonous plant in the phylogenetic tree [38]. Furthermore, the phylogenetic tree also reflects the closer relationship of oats and *A. insularis* to *A. longiglumis*. Intron gain or loss and intron density significantly impact the evolution of large eukaryotic genomes. According to the phylogenetic tree, the *AsHSP90* gene is categorized into six groups, each with a highly similar exon number and exon–intron structure. Similarly, the motif distribution of *AsHSP90* genes across various subgroups exhibits a consistent pattern. Consequently, these analyses further corroborate the reliability of this phylogenetic classification of *AsHSP90* genes.

Numerous studies have demonstrated that the plant HSP90 protein plays a crucial role in responding to various abiotic stresses [39,40]. According to the qRT-PCR results, it was observed that most *AsHSP90* genes were induced under all five stresses, albeit with low expression levels in some genes. HSP is a well-known protein that responds to heat stress and protects plants from high-temperature stress damage [41]. In the current study, the highest expression of all AsHSP90 proteins was observed under high-temperature stress, suggesting that HSP90 is particularly sensitive to heat stress. Group 5 (*AsHSP90-7*, *AsHSP90-8*, and *AsHSP90-9*) within the oat *AsHSP90* gene family exhibited high expression under all five stress conditions. Among them, the *AsHSP90-9* gene, a paralogous gene of *AsHSP90-7* and *AsHSP90-8* replicated, had the highest expression under heat stress. Gene duplication events usually result in an increase in the number of genes; after this, the replicated genes may undergo neofunctionalization or subfunctionalization [42]. In our study, *AsHSP90-9* had similar expression patterns to *AsHSP90-7* and *AsHSP90-8*, and we hypothesized that the *AsHSP90-9* gene may undergo subfunctionalization to carry out some of the functions of the *AsHSP90-7* and *AsHSP90-8* genes. Therefore, under high-temperature stress conditions, the expression of the *AsHSP90-9* genes involved in the response to heat stress after subfunctional differentiation was significantly up-regulated to help the oats better adapt to heat stress. Furthermore, the analysis of the cis-elements of *AsHSP90* in this study revealed a variety of cis-acting elements involved in hormone regulation and abiotic stress. For instance, *AsHSP90-18*, *AsHSP90-19*, and *AsHSP90-19* had the highest number of ABRE-binding sites within the ABA regulatory pathway. ABA-regulated genes are involved in multiple biotic and abiotic stress responses in plants [43]. However, contrary to expectations, these genes did not exhibit the highest expression levels in our RT-qPCR analysis under the five abiotic stress treatments. This discrepancy might be attributed to their status as orthologous genes from different subgenomes, suggesting that their expression is regulated through a complex mechanism of subgenomic homologous co-expression. Thus, the roles of *AsHSP90-9*, *AsHSP90-18*, *AsHSP90-19*, and *AsHSP90-20* in response to these stresses warrant further investigation. Moreover, variations were observed in gene expression at different sites, necessitating further studies to investigate the expression of the AsHSP90 protein in various tissues of oats.

## 4. Materials and Methods

### 4.1. HSP90 Gene Family Identification in Avena sativa, Avena insularis, and Avena longiglumis

The genomic resources of *Avena sativa* and its possible ancestors *Avena insularis* and *Avena longiglumis* were cv “sang_v1.1”, cv “BYU209_v1.1”, and cv “CN58138_v1.1” from the GrainGenes database (https://wheat.pw.usda.gov, accessed on 24 May 2023) [44], and the Hidden Markov Model (HMM) matrix file for the HSP90 structural domain was obtained from the Pfam database (PF00183). Using HMMER software version 3.0 [45], HSP90s were searched for within three genome protein sequences. A BLASTP analysis of these three genomes’ genomic resources was conducted using the protein sequence of the *Brachypodium disachyon* and *Arabidopsis thaliana* HSP90 gene as a query. Based on the HMMER and BLASTP results, all candidate HSP90 proteins that possibly contained the HSP90 domain were submitted to Pfam (http://pfam.xfam.org/, accessed on 24 May 2023) and CDD (https://www.ncbi.nlm.nih.gov/Structure/bwrpsb/bwrpsb.cgi, accessed on 24 May 2023) for confirmation of the final resulted HSP90 members. *A. sativa* HSP90 genes were renamed according to their molecular weight. An in-house Perl script was used to calculate the molecular weight, instability index, and theoretical isoelectric point (pI) of *AsHSP90*. Subcellular localization was predicted using WoLF PSORT [46] (https://wolfpsort.hgc.jp/ accessed on 29 May 2023). All *HSP90* genes were mapped to *A. sativa* chromosomes using TBtools-II [47].

### 4.2. Phylogenetic Analysis of HSP90 Genes

The HSP90 protein sequences of *Avena sativa*, *Avena insularis*, *Avena longiglumis*, *Arabidopsis thaliana*, *Oryza sativa*, *Brachypodium disachyon*, and *Zea mays* were subjected to multiple-sequence alignment analysis using ClustalW [48] in order to study the evolutionary relationship between the HSP90 families of these five plants. A maximum-likelihood (ML) phylogenetic tree was construction by 1000 bootstrapping with MEGA [49].

### 4.3. Structure and Conserved-Motif Analysis of AsHSP90s

To extract the CDS and UTR locations corresponding to *AsHSP90*s, we used in-house Perl scripts. In addition, in the AsHSP90 protein, a phylogenetic tree was constructed using the maximum-likelihood (ML) method and 10 motifs were identified using the MEME program 5.10 [30] (https://meme-suite.org/meme/tools/fimo, accessed on 16 June 2023). The gene structures, motifs, and phylogenetic tree were mapped and modified using TBtools-II [47].

### 4.4. Gene Duplication and Ka and Ks Calculation

Gene duplication search for the identified HSP90 members was performed using blastall. The major criteria used for analyzing potential gene duplications included: (a) length of alignable sequence covers >75% of longer gene, and (b) similarity of aligned regions >75% [50]. The rate of synonymous substitutions (Ka) and nonsynonymous substitutions (Ks) in the *AsHSP90* gene obtained from gene duplication events was calculated using KaKs_Calculator 3.0 [51].

### 4.5. Synteny Analysis of AsHSP90 Genes and Selected Plants

To demonstrate the synteny relationship of the HSP90 genes obtained from *Avena sativa* and gramineous species of *Oryza sativa*, *Brachypodium disachyon*, *A. insularis*, and *A. longiglumis*, the syntenic analysis maps were constructed using the software MCScanX [31]. In addition, gene duplication events in *AsHSP90*s of oats also were visualized with MCScanX. OrthoFinder 2.5.5 [52] was utilized to find directly orthologous genes between the oats and other representative species.

### 4.6. Identification and Analysis of cis-Elements in the Promoter Region of AsHSP90

An in-house Perl script was used to extract 1.5 kb sequences upstream of the *AsHSP90* gene as promoter regions and submit these sequences to PlantCARE [32] for the analysis of cis-regulatory elements.

### 4.7. Three-Dimensional Protein Structure Prediction

Protein templates in the PDB database [53] (https://www.rcsb.org/, accessed on 21 June 2023) with similar 3D structures to the AsHSP90 protein were searched using PSI-BLAST [54], and the resulting templates and AsHSP90 protein sequences were submitted to SWISS-MODEL [33] (https://swissmodel.expasy.org/, accessed on 22 June 2023) for protein 3D structure prediction. Moreover, the 3D model’s quality was assessed with global model quality estimates (GMQE) and QMEAN values. The GMQE scores ranged from 0 to 1, with higher scores indicating a more reliable model, and the QMEAN scores ranged between 0 and −4, with models closer to 0 being of better quality.

### 4.8. Protein–Protein Interaction Network

To predict the interactions between oat HSP90 proteins and related proteins, the AsHSP90 protein sequence was submitted to the STRING database [55]. The organism was set to wheat, and advanced settings were maintained at their default values. PPI networks were visualized using Cytoscape v3.10.1 [56].

### 4.9. Plant Material, Growth Conditions, and Treatment

Viable seeds of oat cv “Baylor” were grown in quartz sand and grown in a greenhouse. The germinated seedlings were transferred into Hoagland’s solution after 7 days. Seedlings were subjected to stress treatments after 14 days. Salt stress was simulated by dissolving a 250 mM concentration of NaCl in the culture broth. Chromium (Cr) (K_2_Cr_2_O_7_) was dissolved at a concentration of 300 mg/L to simulate heavy-metal stress. For heat stress, the temperature was 40 °C during the day/30 °C at night, and the photoperiod was 12 h of light/12 h of dark. Drought stress was stimulated with 20% polyethylene glycol 6000 (PEG) after lysis. Abscisic acid (ABA) was sprayed at a concentration of 100 mM. Live biological replicates of plant leaf samples were collected at 0 h, 3 h, 6 h, 12 h, and 48 h after each application of stress. Then, the stressed seedlings were collected for RNA extraction and stored at a temperature of −8 °C.

### 4.10. RNA Isolation, cDNA Synthesis, and Quantitative Real-Time PCR Expression Analyses

Total RNA was isolated using the Direct-zol™ RNA MiniPrep Kit (Zymo Research, Beijing, China), according to the manufacturer’s protocol. ABScript III RT Master Mix for qPCR with Gdna Remover (Abclonal, Wuhan, China) was used for the synthesis of cDNA. RT-qPCR analyses were performed using the Genious 2× SYBR Green Fast Qpcr Mix (Abclonal, China), in accordance with the manufacturer’s protocol; the reactions were run using the CXF96 Connect™ Real-Time System (Bio-Rad, Singapore). The AsEIF4A gene was used as an internal reference gene to calculate the expression of 20 *AsHSP90* genes [57]. Relative expression was calculated using the 2^−ΔΔCt^ method [58,59]. In addition, twenty primer pairs for the *AsHSP90* gene were designed with Primer 5 software. Primers for the *AsHSP90* gene used in the qRT-PCR assay are listed in Supplementary Table S5.

## 5. Conclusions

We identified and localized 20 *AsHSP90* genes in the oat genome and divided them into six clades. Gene structures and motifs were highly conserved within the same groups. The formation of the *AsHSP90* gene family in oats may have arisen mainly through gene duplication after polyploidy and divergence. Under high-temperature, salt, cadmium (Cd), polyethylene glycol (PEG), and abscisic acid (ABA) stresses, *AsHSP90* showed the strongest expression under heat stress, and members of Group 5 (*AsHSP90-7*, *AsHSP90-8*, and *AsHSP90-9*) were generally highly expressed. The function of AsHSP90 proteins remains unknown, especially the co-expression of homologous proteins among different subgenomes, and further studies are needed to determine their precise function. Our study elucidates the potential pathways for the expansion of the *AsHSP90* gene family in oats, and also lays the foundation for future functional analyses of these AsHSP90 proteins as well as studies of their synergistic expression across subgenomes.

## Figures and Tables

**Table 1:** Biophysical properties and subcellular localization of the oat HSP90 genes.

Gene	ID	Length	MW	pI	Instability Index	Aliphatic Index	GRAVY	Predicted Subcellular Location
AsHSP90-1	AVESA.00010b.r2.5DG0985750.1	627	71,881.4	4.79	41.94	74.32	−0.639	Endoplasmic reticulum
AsHSP90-2	AVESA.00010b.r2.7AG1217140.1	698	80,094.2	4.67	39.68	83.67	−0.592	Cytoplasm
AsHSP90-3	AVESA.00010b.r2.7DG1391180.1	698	80,103.2	4.69	39.7	83.81	−0.597	Chloroplast
AsHSP90-4	AVESA.00010b.r2.5CG0932690.1	699	80,214.3	4.69	39.72	83.98	−0.602	Chloroplast
AsHSP90-5	AVESA.00010b.r2.5AG0850550.1	700	80,432.6	4.67	40.22	82.17	−0.617	Nucleus
AsHSP90-6	AVESA.00010b.r2.5CG0883430.1	700	80,475.6	4.67	40.43	82.03	−0.625	Cytoplasm
AsHSP90-7	AVESA.00010b.r2.5DG0939350.1	707	80,750.8	4.67	40.95	82.48	−0.586	Chloroplast
AsHSP90-8	AVESA.00010b.r2.4CG1254200.1	713	81,379.5	4.67	41.36	82.61	−0.577	Cytoplasm mitochondrion
AsHSP90-9	AVESA.00010b.r2.6AG1070350.1	713	81,411.4	4.62	41.45	82.47	−0.577	Chloroplast
AsHSP90-10	AVESA.00010b.r2.5CG0924830.1	781	88,373.9	4.6	47.64	79.14	−0.531	Nucleus
AsHSP90-11	AVESA.00010b.r2.7DG1384250.1	781	88,429.9	4.58	46.62	80.01	−0.528	Chloroplast
AsHSP90-12	AVESA.00010b.r2.7AG1224100.1	781	88,432	4.6	46.45	79.51	−0.534	Chloroplast
AsHSP90-13	AVESA.00010b.r2.5AG0822120.1	787	88,667.1	5.05	44.63	76.24	−0.565	Cytoplasm
AsHSP90-14	AVESA.00010b.r2.5CG0884240.1	784	88,810.7	4.7	44.1	78.37	−0.544	Cytoplasm
AsHSP90-15	AVESA.00010b.r2.5DG0960220.1	785	89,044.9	4.65	44.92	78.14	−0.553	Endoplasmic reticulum
AsHSP90-16	AVESA.00010b.r2.5AG0849600.1	786	89,235.1	4.69	44.7	78.04	−0.553	Cytoplasm
AsHSP90-17	AVESA.00010b.r2.5CG0914170.1	809	91,165.9	5.09	44.57	78.74	−0.539	Chloroplast
AsHSP90-18	AVESA.00010b.r2.7DG1344440.1	806	92,426.4	4.65	37.35	79.6	−0.703	Cytoplasm
AsHSP90-19	AVESA.00010b.r2.2AG0260460.1	806	92,568.6	4.63	37.28	80.57	−0.69	Chloroplast
AsHSP90-20	AVESA.00010b.r2.6CG1147820.1	806	92,623.6	4.6	37.68	80.09	−0.699	Endoplasmic reticulum

**Table 2:** Segmental duplications of *AsHSP90* paralogous pairs in oats.

Paralogous HSP90 Pairs	chr. Location	Duplication Type	AsHSP90 Group	Ka	Ks	Ka_Ks
AsHSP90-8	chr4C	Segmental	Group 4	0.0036	0.0908	0.0396
AsHSP90-9	chr6A		Group 4			
AsHSP90-7	chr5D	Segmental	Group 4	0.0018	0.1265	0.0143
AsHSP90-8	chr4C		Group 4			
AsHSP90-7	chr5D	Segmental	Group 4	0.0042	0.1343	0.0316
AsHSP90-9	chr6A		Group 4			
AsHSP90-12	chr7A	Segmental	Group 3	0.0016	0.0234	0.0706
AsHSP90-11	chr7D		Group 3			
AsHSP90-12	chr7A	Segmental	Group 3	0.0080	0.0928	0.0862
AsHSP90-10	chr5C		Group 3			
AsHSP90-12	chr7A	Segmental	Group 3	0.0638	0.9539	0.0669
AsHSP90-15	chr5D		Group 3			
AsHSP90-12	chr7A	Segmental	Group 3	0.0656	0.9452	0.0694
AsHSP90-14	chr5C		Group 3			
AsHSP90-11	chr7D	Segmental	Group 3	0.0097	0.0798	0.1211
AsHSP90-10	chr5C		Group 3			
AsHSP90-11	chr7D	Segmental	Group 3	0.0647	0.9356	0.0691
AsHSP90-15	chr5D		Group 3			
AsHSP90-11	chr7D	Segmental	Group 3	0.0665	0.9406	0.0707
AsHSP90-14	chr5C		Group 3			

## Data Availability

Data are contained within the article and Supplementary Materials.

## Supplementary Materials

The following supporting information can be downloaded at: https://www.mdpi.com/article/10.3390/ijms25042305/s1.

## References

[1] Guihur A., Rebeaud M.E., Goloubinoff P. (2022). How Do Plants Feel the Heat and Survive?. Trends Biochem. Sci..

[2] Zhang H., Zhu J., Gong Z., Zhu J.-K. (2022). Abiotic Stress Responses in Plants. Nat. Rev. Genet..

[3] Li B., Gao K., Ren H., Tang W. (2018). Molecular Mechanisms Governing Plant Responses to High Temperatures. J. Integr. Plant Biol..

[4] Oldroyd G.E.D., Leyser O. (2020). A Plant’s Diet, Surviving in a Variable Nutrient Environment. Science.

[5] Tigchelaar M., Battisti D.S., Naylor R.L., Ray D.K. (2018). Future Warming Increases Probability of Globally Synchronized Maize Production Shocks. Proc. Natl. Acad. Sci. USA.

[6] Gray S.B., Brady S.M. (2016). Plant Developmental Responses to Climate Change. Dev. Biol..

[7] Wen J., Qin Z., Sun L., Zhang Y., Wang D., Peng H., Yao Y., Hu Z., Ni Z., Sun Q. (2023). Alternative Splicing of TaHSFA6e Modulates Heat Shock Protein-Mediated Translational Regulation in Response to Heat Stress in Wheat. New Phytol..

[8] di Donato M., Geisler M. (2019). HSP90 and Co-Chaperones: A Multitaskers’ View on Plant Hormone Biology. FEBS Lett..

[9] Tichá T., Samakovli D., Kuchařová A., Vavrdová T., Šamaj J. (2020). Multifaceted Roles of HEAT SHOCK PROTEIN 90 Molecular Chaperones in Plant Development. J. Exp. Bot..

[10] Yadav A., Singh J., Ranjan K., Kumar P., Khanna S., Gupta M., Kumar V., Wani S., Sirohi A. (2020). Heat Shock Proteins: Master Players for Heat-Stress Tolerance in Plants during Climate Change. Heat Stress Tolerance in Plants: Physiological, Molecular and Genetic Perspectives.

[11] Genest O., Wickner S., Doyle S.M. (2019). Hsp90 and Hsp70 Chaperones: Collaborators in Protein Remodeling. J. Biol. Chem..

[12] Chiosis G., Digwal C.S., Trepel J.B., Neckers L. (2023). Structural and Functional Complexity of HSP90 in Cellular Homeostasis and Disease. Nat. Rev. Mol. Cell Biol..

[13] Dutta R., Inouye M. (2000). GHKL, an Emergent ATPase/Kinase Superfamily. Trends Biochem. Sci..

[14] Picard D. (2002). Heat-Shock Protein 90, a Chaperone for Folding and Regulation. Cell. Mol. Life Sci. CMLS.

[15] Hahn A., Bublak D., Schleiff E., Scharf K.-D. (2011). Crosstalk between Hsp90 and Hsp70 Chaperones and Heat Stress Transcription Factors in Tomato. Plant Cell.

[16] Li W., Chen Y., Ye M., Wang D., Chen Q. (2020). Evolutionary History of the Heat Shock Protein 90 (Hsp90) Family of 43 Plants and Characterization of Hsp90s in Solanum Tuberosum. Mol. Biol. Rep..

[17] Krishna P., Gloor G. (2001). The Hsp90 Family of Proteins in Arabidopsis Thaliana. Cell Stress Chaperones.

[18] Zhang M., Shen Z., Meng G., Lu Y., Wang Y. (2017). Genome-Wide Analysis of the *Brachypodium Distachyon* (L.) P. Beauv. Hsp90 Gene Family Reveals Molecular Evolution and Expression Profiling under Drought and Salt Stresses. PLoS ONE.

[19] Song Z., Pan F., Yang C., Jia H., Jiang H., He F., Li N., Lu X., Zhang H. (2019). Genome-Wide Identification and Expression Analysis of HSP90 Gene Family in Nicotiana Tabacum. BMC Genet..

[20] Appiah C., Yang Z.-F., He J., Wang Y., Zhou J., Xu W.-Z., Nie G., Zhu Y.-Q. (2021). Genome-Wide Identification of Hsp90 Gene Family in Perennial Ryegrass and Expression Analysis under Various Abiotic Stresses. Plants.

[21] Song H., Zhao R., Fan P., Wang X., Chen X., Li Y. (2009). Overexpression of AtHsp90.2, AtHsp90.5 and AtHsp90.7 in Arabidopsis Thaliana Enhances Plant Sensitivity to Salt and Drought Stresses. Planta.

[22] Liu Y., Burch-Smith T., Schiff M., Feng S., Dinesh-Kumar S.P. (2004). Molecular Chaperone Hsp90 Associates with Resistance Protein N and Its Signaling Proteins SGT1 and Rar1 to Modulate an Innate Immune Response in Plants. J. Biol. Chem..

[23] Hubert D.A., Tornero P., Belkhadir Y., Krishna P., Takahashi A., Shirasu K., Dangl J.L. (2003). Cytosolic HSP90 Associates with and Modulates the Arabidopsis RPM1 Disease Resistance Protein. EMBO J..

[24] Banilas G., Korkas E., Englezos V., Nisiotou A.A., Hatzopoulos P. (2012). Genome-Wide Analysis of the Heat Shock Protein 90 Gene Family in Grapevine (*Vitis Vinifera* L.). Aust. J. Grape Wine Res..

[25] Samakovli D., Roka L., Dimopoulou A., Plitsi P.K., Žukauskait A., Georgopoulou P., Novák O., Milioni D., Hatzopoulos P. (2021). HSP90 Affects Root Growth in Arabidopsis by Regulating the Polar Distribution of PIN1. New Phytol..

[26] Baum B.R. (1977). Oats: Wild and Cultivated. A Monograph of the Genus Avena L. (Poaceae).

[27] Fu J., Zhang Y., Hu Y., Zhao G., Tang Y., Zou L. (2020). Concise Review: Coarse Cereals Exert Multiple Beneficial Effects on Human Health. Food Chem..

[28] Oats|Diseases and Pests, Description, Uses, Propagation. https://plantvillage.psu.edu/topics/oats/infos.

[29] Kamal N., Tsardakas Renhuldt N., Bentzer J., Gundlach H., Haberer G., Juhász A., Lux T., Bose U., Tye-Din J.A., Lang D. (2022). The Mosaic Oat Genome Gives Insights into a Uniquely Healthy Cereal Crop. Nature.

[30] Bailey T.L., Boden M., Buske F.A., Frith M., Grant C.E., Clementi L., Ren J., Li W.W., Noble W.S. (2009). MEME SUITE: Tools for Motif Discovery and Searching. Nucleic Acids Res..

[31] Wang Y., Tang H., Debarry J.D., Tan X., Li J., Wang X., Lee T., Jin H., Marler B., Guo H. (2012). MCScanX: A Toolkit for Detection and Evolutionary Analysis of Gene Synteny and Collinearity. Nucleic Acids Res..

[32] Rombauts S., Déhais P., Van Montagu M., Rouzé P. (1999). PlantCARE, a Plant Cis-Acting Regulatory Element Database. Nucleic Acids Res..

[33] Waterhouse A., Bertoni M., Bienert S., Studer G., Tauriello G., Gumienny R., Heer F.T., de Beer T.A.P., Rempfer C., Bordoli L. (2018). SWISS-MODEL: Homology Modelling of Protein Structures and Complexes. Nucleic Acids Res..

[34] Zhang J., Li J., Liu B., Zhang L., Chen J., Lu M. (2013). Genome-Wide Analysis of the Populus Hsp90 Gene Family Reveals Differential Expression Patterns, Localization, and Heat Stress Responses. BMC Genom..

[35] Peng Y., Yan H., Guo L., Deng C., Wang C., Wang Y., Kang L., Zhou P., Yu K., Dong X. (2022). Reference Genome Assemblies Reveal the Origin and Evolution of Allohexaploid Oat. Nat. Genet..

[36] Zhang Y., Zheng L., Yun L., Ji L., Li G., Ji M., Shi Y., Zheng X. (2022). Catalase (CAT) Gene Family in Wheat (*Triticum Aestivum* L.): Evolution, Expression Pattern and Function Analysis. Int. J. Mol. Sci..

[37] Delsuc F., Brinkmann H., Philippe H. (2005). Phylogenomics and the Reconstruction of the Tree of Life. Nat. Rev. Genet..

[38] Radoeva T., Vaddepalli P., Zhang Z., Weijers D. (2019). Evolution, Initiation, and Diversity in Early Plant Embryogenesis. Dev. Cell.

[39] Pearl L.H., Prodromou C. (2006). Structure and Mechanism of the Hsp90 Molecular Chaperone Machinery. Annu. Rev. Biochem..

[40] Reddy R.K., Chaudhary S., Patil P., Krishna P. (1998). The 90 kDa Heat Shock Protein (Hsp90) Is Expressed throughout Brassica Napus Seed Development and Germination. Plant Sci..

[41] Ul Haq S., Khan A., Ali M., Khattak A.M., Gai W.-X., Zhang H.-X., Wei A.-M., Gong Z.-H. (2019). Heat Shock Proteins: Dynamic Biomolecules to Counter Plant Biotic and Abiotic Stresses. Int. J. Mol. Sci..

[42] Blanc G., Wolfe K.H. (2004). Widespread Paleopolyploidy in Model Plant Species Inferred from Age Distributions of Duplicate Genes. Plant Cell.

[43] Nakashima K., Yamaguchi-Shinozaki K. (2013). ABA Signaling in Stress-Response and Seed Development. Plant Cell Rep..

[44] O’Sullivan H. (2007). GrainGenes. Methods Mol. Biol..

[45] Potter S.C., Luciani A., Eddy S.R., Park Y., Lopez R., Finn R.D. (2018). HMMER Web Server: 2018 Update. Nucleic Acids Res..

[46] Horton P., Park K.-J., Obayashi T., Fujita N., Harada H., Adams-Collier C.J., Nakai K. (2007). WoLF PSORT: Protein Localization Predictor. Nucleic Acids Res..

[47] Chen C., Wu Y., Li J., Wang X., Zeng Z., Xu J., Liu Y., Feng J., Chen H., He Y. (2023). TBtools-II: A “One for All, All for One” Bioinformatics Platform for Biological Big-Data Mining. Mol. Plant.

[48] Thompson J.D., Higgins D.G., Gibson T.J. (1994). CLUSTAL W: Improving the Sensitivity of Progressive Multiple Sequence Alignment through Sequence Weighting, Position-Specific Gap Penalties and Weight Matrix Choice. Nucleic Acids Res..

[49] Kumar S., Stecher G., Li M., Knyaz C., Tamura K. (2018). MEGA X: Molecular Evolutionary Genetics Analysis across Computing Platforms. Mol. Biol. Evol..

[50] Gu Z., Cavalcanti A., Chen F.-C., Bouman P., Li W.-H. (2002). Extent of Gene Duplication in the Genomes of Drosophila, Nematode, and Yeast. Mol. Biol. Evol..

[51] Zhang Z. (2022). KaKs_Calculator 3.0: Calculating Selective Pressure on Coding and Non-Coding Sequences. Genom. Proteom. Bioinform..

[52] Emms D.M., Kelly S. (2019). OrthoFinder: Phylogenetic Orthology Inference for Comparative Genomics. Genome Biol..

[53] Berman H.M., Westbrook J., Feng Z., Gilliland G., Bhat T.N., Weissig H., Shindyalov I.N., Bourne P.E. (2000). The Protein Data Bank. Nucleic Acids Res..

[54] Bhagwat M., Aravind L. (2007). PSI-BLAST Tutorial. Methods Mol. Biol..

[55] Szklarczyk D., Gable A.L., Lyon D., Junge A., Wyder S., Huerta-Cepas J., Simonovic M., Doncheva N.T., Morris J.H., Bork P. (2019). STRING V11: Protein-Protein Association Networks with Increased Coverage, Supporting Functional Discovery in Genome-Wide Experimental Datasets. Nucleic Acids Res..

[56] Shannon P., Markiel A., Ozier O., Baliga N.S., Wang J.T., Ramage D., Amin N., Schwikowski B., Ideker T. (2003). Cytoscape: A Software Environment for Integrated Models of Biomolecular Interaction Networks. Genome Res..

[57] Yang Z., Wang K., Aziz U., Zhao C., Zhang M. (2020). Evaluation of Duplicated Reference Genes for Quantitative Real-Time PCR Analysis in Genome Unknown Hexaploid Oat (*Avena Sativa* L.). Plant Methods.

[58] Pandey A., Khan M.K., Hamurcu M., Brestic M., Topal A., Gezgin S. (2022). Insight into the Root Transcriptome of a Boron-Tolerant Triticum Zhukovskyi Genotype Grown under Boron Toxicity. Agronomy.

[59] Schmittgen T.D., Livak K.J. (2008). Analyzing Real-Time PCR Data by the Comparative C(T) Method. Nat. Protoc..

