# Supplementary material for: Genome-Wide Analysis of the Oat (Avena sativa) HSP90 Gene Family Reveals Its Identification, Evolution, and Response to Abiotic Stress
Source: Int J Mol Sci. 2024 Feb 15;25(4):2305. doi: 10.3390/ijms25042305 (PMC10889330; doi:10.3390/ijms25042305)
Supplement: Supplementary file 1 [file ijms-25-02305-s001.zip › ijms-2805599-supplementary/Supplementary data/Supplementary Figure S3.pdf]

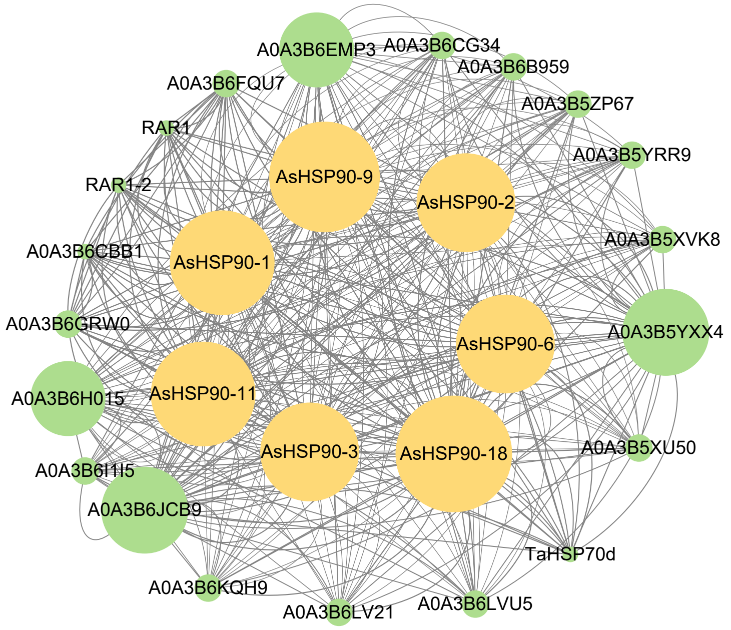

Supplementary figure 3. Protein–protein interaction (PPI) networks of AsHSP90s and their related proteins. The protein node size represents the interaction degrees linked with each node.
