# Supplementary figures and images for: Genome-Wide Analysis of the Oat (Avena sativa) HSP90 Gene Family Reveals Its Identification, Evolution, and Response to Abiotic Stress
Source: Int J Mol Sci. 2024 Feb 15;25(4):2305. doi: 10.3390/ijms25042305 (PMC10889330; doi:10.3390/ijms25042305)

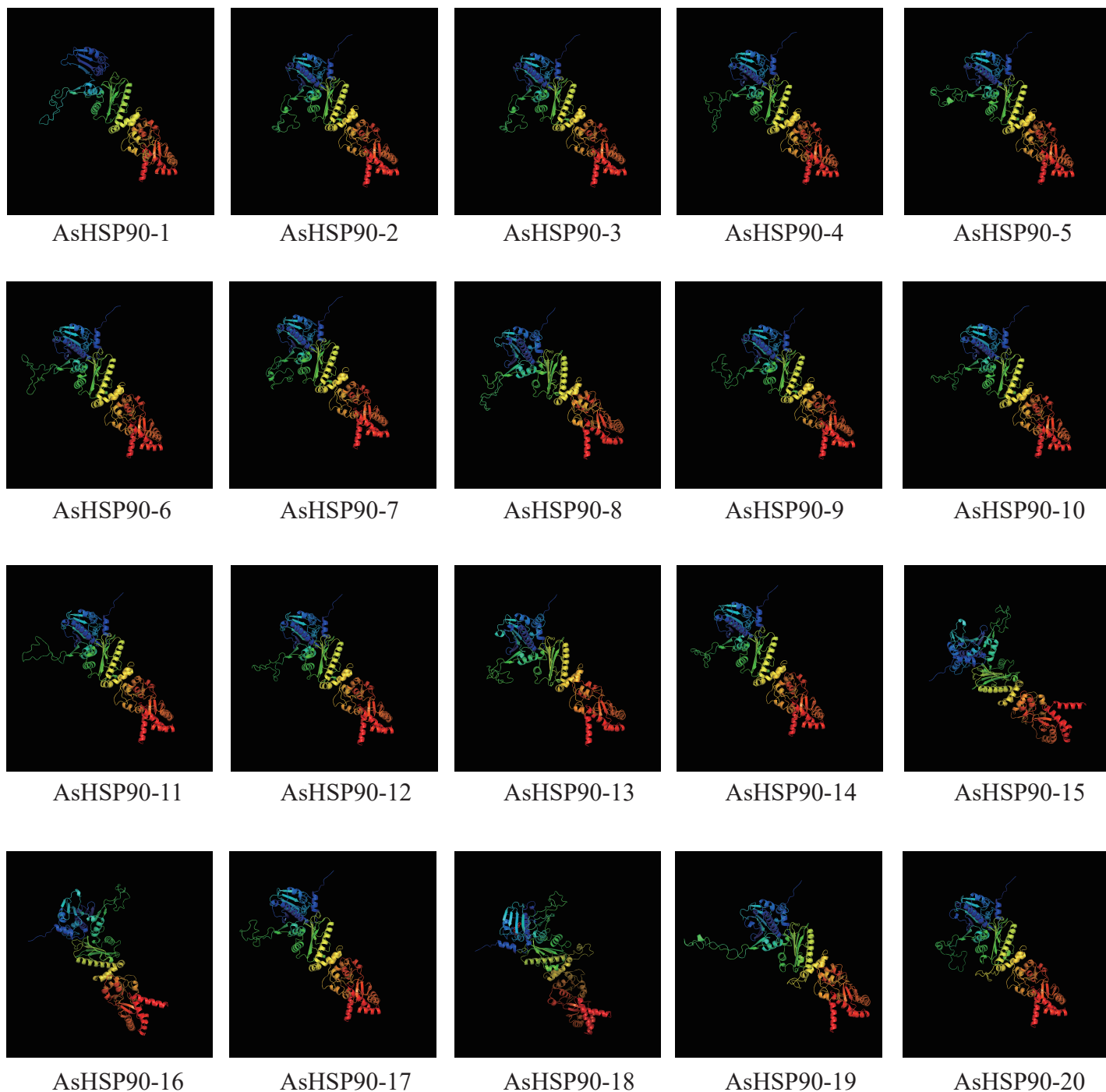

Supplementary figure2. Three-dimensional (3D) protein structures of AsHSP90s.

Supplement: Supplementary file 1 [file ijms-25-02305-s001.zip › ijms-2805599-supplementary/Supplementary data/Supplementary Figure S2.pdf]
